# The role of the retinal vasculature in age-related macular degeneration: a spotlight on OCTA

**DOI:** 10.1038/s41433-023-02721-7

**Published:** 2023-09-06

**Authors:** Thomas R. P. Taylor, Martin J. Menten, Daniel Rueckert, Sobha Sivaprasad, Andrew J. Lotery

**Affiliations:** 1 Clinical and Experimental Sciences, Faculty of Medicine, University of Southampton, Southampton, UK; 2 BioMedIA, Imperial College London, London, UK; 3 Institute for AI and Informatics in Medicine, Klinikum Rechts der Isar, Technical University Munich, Munich, Germany; 4 NIHR Moorfields Biomedical Research Centre, Moorfields Eye Hospital NHS Foundation Trust, London, UK

**Keywords:** Predictive markers, Haemic and immune systems

## Abstract

Age-related macular degeneration (AMD) remains a disease with high morbidity and an incompletely understood pathophysiological mechanism. The ocular blood supply has been implicated in the development of the disease process, of which most research has focused on the role of the choroid and choriocapillaris. Recently, interest has developed into the role of the retinal vasculature in AMD, particularly with the advent of optical coherence tomography angiography (OCTA), which enables non-invasive imaging of the eye’s blood vessels. This review summarises the up-to-date body of work in this field including the proposed links between observed changes in the retinal vessels and the development of AMD and potential future directions for research in this area. The review highlights that the strongest evidence supports the observation that patients with early to intermediate AMD have reduced vessel density in the superficial vascular complex of the retina, but also emphasises the need for caution when interpreting such studies due to their variable methodologies and nomenclature.

## Abstract (zh)

老年黄斑变性(AMD)仍为高患病率, 病理生理机制尚未完全阐明的疾病。眼部供血与疾病的进展有关, 大多数研究集中在脉络膜和脉络膜毛细血管的作用。最近, 人们对视网膜血管在AMD发病中的作用产生了兴趣, 尤其是随着光学相干断层扫描血管造影(OCTA)的出现, 可对视网膜血管进行无创成像。本综述总结了该领域的最新工作, 包括视网膜血管的病理生理性改变与AMD进展之间的相关性, 以及该领域未来研究的潜在方向。本综述强调了极有力的证据支持早期至中期AMD患者视网膜表层血管密度降低的观察结果, 但也强调由于这些研究使用的方法和命名方式不同, 在阐释这些研究结果时需要谨慎。

## Introduction

Age-related macular degeneration (AMD) is a common degenerative condition affecting the macula. It causes a progressive distortion of central vision and is one of the leading causes of visual impairment in the elderly, with a markedly increased prevalence in older age groups [1]. AMD is characterised in the early phase of the disease by retinal pigment epithelium (RPE) abnormalities and accumulation of drusen between the RPE and Bruch’s membrane.

AMD severity is typically graded according to the Beckman classification [2]. This defines early AMD as persons with medium sized drusen (63–124 μm) without pigmentary abnormalities and intermediate AMD as persons with large drusen (125 μm or larger), or those with medium drusen and pigmentary change. Late-stage AMD manifests itself as two types; geographic atrophy and neovascular (or “wet”, or exudative) AMD. Geographic atrophy involves irreversible degeneration of the photoreceptors, RPE and choriocapillaris layers whereas neovascular AMD involves abnormal growth of choroidal blood vessels through Bruch’s membrane and subsequent fluid leakage, bleeding and scarring.

Despite extensive research, the pathogenesis of AMD is incompletely understood and consequently interventions to reduce the risk of disease progression from early to advanced stages are limited to lifestyle modifications and nutrient preparations, either through diet or oral supplements. A prominent school of thought has focused on the role of the complement cascade and in particular regulatory complements, complement factors H and I, and complement C3 and its downstream pathway [3, 4]. Aberrant complement activation is thought to trigger a chronic low-grade inflammation at the macula which leads to damage to the retina and choroid. Generation of reactive oxygen species by the photoreceptor-RPE complex and accumulation of lipids surrounding the RPE have also been implicated [5].

More recently, there has been an increased interest in the role of the vasculature in the pathogenesis of AMD and the way in which differences in blood supply to retinal tissues may affect tissue damage and disease progression. This has often been centred on the role of the choroid and specifically the choriocapillaris and this has been well summarised elsewhere [6, 7]. A less extensively researched, but developing, area of interest is in the retinal vasculature and to the authors’ knowledge, no comprehensive review of the body of literature in this area yet exists. This review seeks therefore to summarise the current research insights into the contribution of the retinal vasculature to the pathogenesis of AMD. The anatomy of the retinal vasculature is discussed, followed by a summary of studies investigating this area, both before and after the development of optical coherence tomography angiography (OCTA) which substantially improved imaging of the retinal vessels.

This is a narrative review, conducted through an initial search of the PubMed database for “retinal vessel” or “retinal vasc*” with “AMD”. Individual case reports were not included but there was otherwise no lower limit of the number patients included in each study. Articles were not restricted by publication date but were limited to English language publications. This was combined with a review of references and citations from relevant articles for completeness, after initial screening. The initial search strategy did not attempt to specify OCTA studies but care was taken on review of references to ensure all relevant material was included.

## Anatomy of the retinal vasculature

The blood supply to the retina arises from the ophthalmic artery, the first branch of the internal carotid artery [8]. Branches of the ophthalmic artery then form two complementary systems that supply different retinal layers and the choroid. Multiple branches become the short posterior ciliary arteries which enter through the sclera outside the optic nerve. These short posterior ciliary arteries supply the multi-layered choroidal circulation, of which the closest layer to the retina is the choriocapillaris, a dense capillary network adjacent to Bruch’s membrane. The vessels of the choriocapillaris are fenestrated and allow passage of oxygen and nutrients to supply the RPE and the photoreceptor layer constituting the outer one third of the retina.

**Fig. 1:**
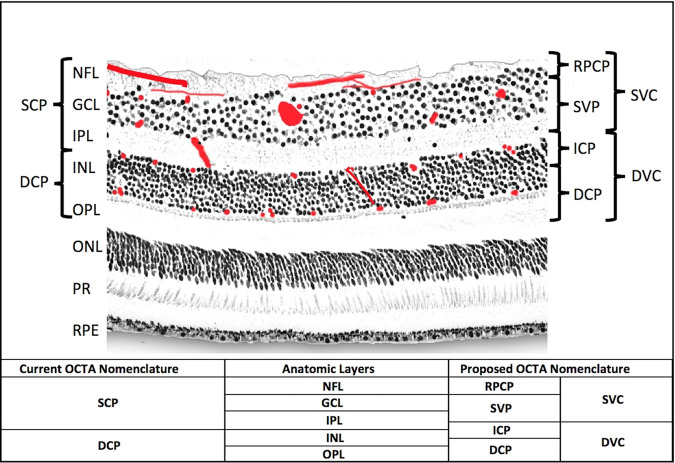
An illustration of the retinal vascular plexuses in red (labelled on right) hand drawn on top of a histological section of the human retina showing anatomic layers (labelled on left) from spectral domain optical coherence tomography. The four vascular plexuses can be grouped into superficial and deep vascular complexes (SVC and DVC, as shown on right) for routine segmentation, but ought to reflect the anatomic location of the ICP at the IPL/INL interface, which the current OCTA segmentations use as a border between superficial and deep plexuses (labelled on left as SCP and DCP). Current and proposed vascular nomenclature and OCTA segmentations are shown at the bottom. (NFL nerve fibre layer, GCL ganglion cell layer, IPL inner plexiform layer, INL inner nuclear layer, OPL outer plexiform layer plus Henle’s fibre layer, ONL outer nuclear layer, PR photoreceptor layers, RPE retinal pigment epithelium, OCTA optical coherence tomography angiography, RPCP radial peripapillary capillary plexus, SVP superficial vascular plexus, ICP intermediate capillary plexus, DCP deep capillary plexus). This figure is reproduced from Campbell et al. [11], under the Creative Commons Attribution 4.0 International License. No changes to content were made. http://creativecommons.org/licenses/by/4.0/.

The inner two thirds of the retina are supplied by the central retina artery (CRA), another branch of the ophthalmic artery, which passes into the eye within the optic nerve. The CRA divides into two superior and two inferior branches that supply a quadrant of the retina each. These vessels run in the retinal nerve fibre layer beneath the internal limiting membrane and give rise to four distinct diffuse capillary networks on histology [9, 10], which are illustrated in Fig. 1. The innermost network is the superficial vascular plexus (SVP), which is located mainly in the ganglion cell layer. Below this are the intermediate capillary plexus (ICP), which lies in between the inner plexiform layer and the inner nuclear layer, and the deep capillary plexus (DCP), which is located in the outer portion of the inner nuclear layer and the inner portion of the outer plexiform layer [11]. The ICP and the DCP are supplied by vertical anastomoses from the SVP. These capillary layers become denser nearer the macula, but are absent over the fovea centralis in an area known as the foveal avascular zone (FAZ). There also exists a fourth capillary network supplied by the CRA called the radial peripapillary capillary plexus (RPCP) which surrounds the optic nerve and the vessels are closely associated with the nerve fibre axons. There are often inconsistencies between studies in the terminology used to refer to the layers of the retinal vasculature. This is described in more detail later in the review along with justification for using the above nomenclature.

Venous drainage of ocular blood closely relates to the arterial system. After passage through the capillary plexuses, inner retinal blood flows back along retinal venules and into the central retinal vein. The choroidal circulation drains into vortex veins which in turn flow into the superior and inferior orbital veins.

## Investigating the role of the retinal vasculature in AMD prior to OCTA

Early attempts to investigate blood flow to the retina in patients with AMD in vivo focused on the use of colour doppler imaging to measure blood flow in ocular vessels. Friedman et al. [12] were able to record pulsatility indices and blood flow velocity from a number of vessels supplying the eye. They showed that eyes with AMD have increased pulsatility in the central retinal artery, as well as in the ciliary arteries, when compared to healthy controls and suggest that this could indicate increased resistance in the capillary beds that they supply. Ciulla et al. [13] in a similar study also detected a lower end diastolic velocity and a higher resistance index in the central retinal arteries of patients with AMD and suggest that there may be a more generalised perfusion abnormality beyond the choroid in these patients.

Other studies have looked at vessel characteristics on colour fundus photographs and have yielded mixed results. Several prospective cohort studies investigating incident AMD in Dutch, Australian and Chinese populations found no relationship between structural differences in arteriolar or venule characteristics and the development of AMD [14–16]. A Malay cohort found wider venule calibre to be associated with early AMD [17], whereas a separate Chinese study, this one in a rural cohort, found increased retinal arteriolar calibre to be associated with early AMD [18]. A study in patients with Acquired Immunodeficiency Syndrome (AIDS) found that both arterioles and venules were more dilated in patients with AMD than without [19], and an American study found that a smaller arteriole to venule diameter ratio was correlated with more advanced AMD [20].

The lack of consistency in these studies may be related to a number of factors including variable stages of AMD being investigated, different methods of controlling for other factors that may independently affect vessel calibre and different study population characteristics. Furthermore, colour fundus photography is limited in its ability to resolve smaller retinal vessels and the deeper vasculature. Although there have been recent attempts to improve these methods of vessel analysis through automation and machine learning [21], use of these relatively crude measures of the retinal vasculature has largely been superseded in recent years by the development of OCTA.

## OCTA and the role of the retinal vasculature in AMD

**Fig. 2:**
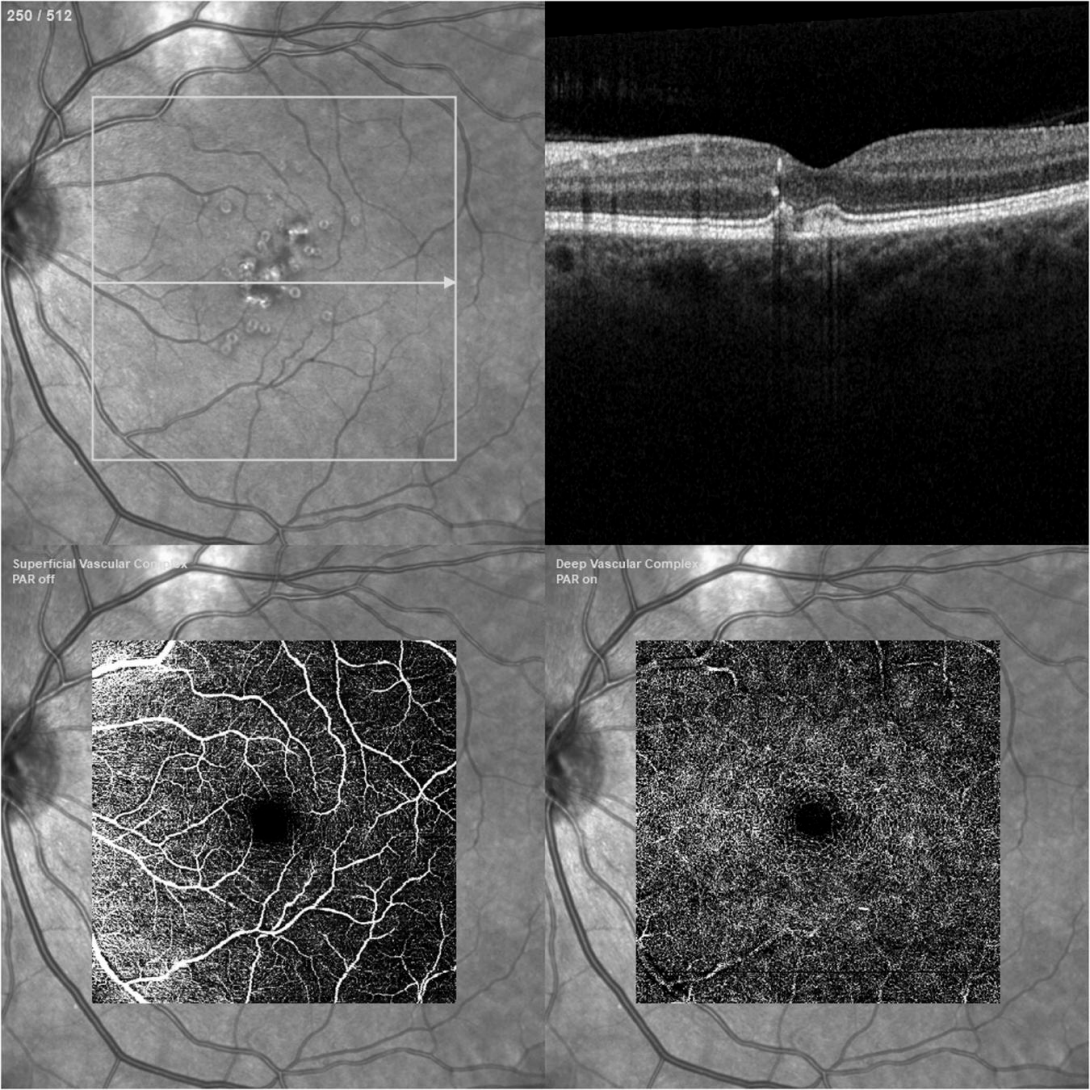
Retinal imaging of a patient with intermediate age-related macular degeneration: red-free (top left), structural OCT (top right), OCT angiography of superficial vascular complex (bottom left), OCT angiography of deep vascular complex (bottom right). Images taken with Spectralis OCT Angiography Module, Heidelberg Engineering Ltd, Hemel Hempstead, UK.

Optical coherence tomography (OCT) was described in the early 1990s and revolutionised ophthalmic imaging. By directing a low-coherence light beam at the sample and measuring the interference of the reflected signal and a reference beam, OCT is able to provide a high-resolution, cross-sectional view of the retina in a fast, non-invasive and dye-independent manner [22]. OCT angiography (OCTA) extends this technology to allow the detection of temporal changes in the sample by acquiring sequential scans of the same region and measuring the decorrelation of these [23]. Most retinal structures are static and appear dark in these images but the light beam is reflected by red blood cells moving freely within the blood vessels and this results in the blood vessels appearing bright in OCTA images, allowing the creation of a map of the retinal vasculature. An example of OCTA imaging is shown in Fig. 2. The most common and commercially available type of OCTA is spectral-domain (SD-OCTA) however some centres have access to swept-source OCTA (SS-OCTA) which can also, to a variable degree, display flow in the choriocapillaris and choroidal vessels. Imaging software allows these vascular maps to be displayed *en-face* and at adjustable retinal depths so that blood flow in different retinal layers and corresponding to individual vascular plexuses can be assessed.

When discussing individual studies in the next section, we use the terminology that each paper uses when referring to their vascular regions and OCTA metrics of interest in order to avoid inadvertently distorting their results. The implications of this are discussed in a later section, as are theories about how differences in the retinal vasculature might affect AMD pathogenesis.

### OCTA in early- and intermediate-stage AMD

OCTA has become widely available commercially during the last decade and the number of clinical studies utilising it to investigate a variety of retinal and choroidal pathologies has substantially increased. The earliest study of the retinal vasculature in AMD using OCTA was by Toto et al. [24]. They ignited interest in the use of this technology to advance insights into the pathophysiology of AMD by looking at whether the capillary plexuses within the retina are altered in patients with the disease. Their study evaluated the vessel density in what they refer to as the “superficial and deep retinal plexuses” between patients with early and intermediate AMD (iAMD) versus a healthy control group. Taking “patients with AMD” as a whole, they found significantly reduced vessel densities in both superficial and deep plexuses in this group versus their controls. Considering the groups separately, the superficial plexus density was significantly reduced in iAMD specifically versus controls, and there was a non-significant reduction in deep plexus density.

Other studies have since also measured changes to vessel density in their own cohorts of non-late-stage AMD. Toto et al. [25] performed a follow-up study on patients with iAMD divided into two groups based on the presence or absence of OCT changes known to precede drusen-associated atrophy (outer plexiform layer (OPL) and inner nuclear layer subsidence and presence of a hyporeflective wedge-shaped band within the limits of the OPL) and compared to healthy controls. In this case, they found a significant reduction in the flow density of the parafoveal superficial vascular plexus (SVP) in patients with iAMD and pre-atrophic changes, compared to both iAMD patients without pre-atrophic changes, and controls. They did not find a significant difference in flow density between iAMD patients without pre-atrophic changes and healthy controls in contrast to their previous study, nor differences in flow densities of the deep vascular plexus. Toto et al.’s use of “flow density” is synonymous with other reports of “vessel density” and highlights inconsistencies in terminology in this field.

Lee et al. [26] found significant differences in the vessel density of both the superficial and deep capillary plexuses between eyes with early AMD and eyes with no evidence of AMD, although it should be noted that both these groups were the fellow eyes of patients with neovascular AMD. Cicenilli et al. [27] did not select a specific AMD stage but stratified their patients by presence of reticular pseudodrusen (RPD), RPD + outer retinal atrophy (ORA), and drusen. They found significantly reduced vessel density of the superior capillary plexus in groups with RPD, and RPD + ORA versus healthy eyes, and also reduced vessel density of the deep capillary plexus in all study groups versus controls. Trinh et al. [28] in their first study in this area also found significantly reduced vessel density of the superior capillary plexus in eyes with iAMD versus controls which was most pronounced in the superior portion of the macula, as well as a non-significant decrease for the deep capillary plexus. They additionally found differences in vessel length, diameter and complexity (determined by dividing the square of the total vessel perimeter over 4*π*vascular density) between the groups. Ozcaliskan et al. [29] found the vessel density of the parafoveal, but not central foveal, superficial capillary plexus to be significantly reduced in iAMD eyes versus healthy controls, which was most pronounced in the superior macula. Shin et al. [30] also found the superficial capillary plexus to be affected in a mixed group of patients with early, intermediate and non-foveal atrophy AMD patients.

One of the most up to date studies in this area is a second paper by Trinh et al. [31], which attempted to characterise whether the retinal vasculature was uniformly altered in AMD or whether the changes were location specific. They used a technique called “spatial clustering” to compare vascular perfusion in different areas of the retina between patients with iAMD and healthy controls. They demonstrated a more pronounced perfusion density reduction in the temporal retina and FAZ in the superficial vascular complex of iAMD patients, and relative sparing of the nasal macula suggesting that the radial peripapillary capillary plexus is less affected at this stage of the disease. They also found that perfusion density was reduced in the deep vascular complex, particularly at the FAZ and diffusely elsewhere.

Not all studies have been in agreement however, with Vaghefi [32] and Parisi [33] both finding no significant differences in retinal vessel density between eyes with iAMD and healthy eyes.

### OCTA in late-stage AMD

Two studies have looked at changes in the retinal vasculature in late-stage AMD. You et al. [34] found significantly reduced vessel density in the superficial vascular complex, intermediate capillary plexus and deep capillary plexus of eyes with geographic atrophy compared to healthy eyes. Qiu et al. [35] on the other hand looked at wet AMD and found a significant reduction in vessel density in the superficial and deep capillary plexus in eyes with neovascular changes versus healthy controls although they do not specify whether they include the neovascular membrane in their vessel density calculations. This effect was also true for the non-neovascular fellow eye compared to controls. Both these studies suggest that the retinal vasculature may be affected in late-stage AMD however further analysis by You et al. [34] found that the significant difference in vascular density was present within areas of GA when compared to healthy eyes, but not in regions outside of the areas of atrophy. This could therefore imply that the changes in the retinal vasculature are caused by, rather than are a cause of, the atrophy.

### OCTA within AMD groups

Additional studies report observations on differences in vessel density at different stages of AMD. Lee et al. [36] found that vessel density in the superficial capillary plexus was reduced in eyes with exudative AMD compared to eyes with non-exudative AMD although this finding was not repeated by Can et al. [37] who found no significant difference, albeit in a smaller cohort. Additionally, Ahn et al. [38] showed that vessel density does not differ among eyes with AMD with and without reticular pseudodrusen.

To date, almost all studies in this area have been cross-sectional in their design. Reiter et al. [39] performed one of the only longitudinal studies and looked at vessel density of the superficial and deep capillary plexuses in patients with iAMD at a 12 month interval and found no significant difference in their measurements over this time period. Ongoing longitudinal studies investigating the progression of iAMD such as the PINNACLE study [40] may provide valuable data to supplement this field in future.

### Other OCTA metrics

Table 1 provides a list of all relevant studies published to date in this area. This review has so far focussed on vessel density as the primary metric of interest as it is most commonly reported in the literature however it is not the only quantitative measure used to assess OCTA scans of the retina. Foveal avascular zone (FAZ) area has long been observed to be increased in patients with diabetic retinopathy [41] and various studies previously discussed also attempted to look at FAZ characteristics in AMD patients. The majority of studies have found no evidence of differences in FAZ parameters including area, perimetry and circularity indices between AMD patients and controls, and within AMD groups [26–29, 32, 36, 37, 42] with only two studies finding a reduced circularity index in AMD patients [30, 36] and one finding an enlarged FAZ area in AMD patients [30].

**Table 1:** Studies using OCT-A to investigate the retinal vasculature in age-related macular degeneration (AMD).

Study	Year	Stages of AMD investigated	Number of AMD eyes	Comparators	Comparator eyes	Vascular anatomy investigated	OCT-A and OCT indices used	OCT-A device used
Toto	2016	Early, Intermediate	14, 23	Healthy Controls	21	Superficial Retinal Plexus, Deep Retinal Plexus	Vessel Density, Choroidal Thickness	XR Avanti AngioVue OCTA (Optovue Inc, Fremont, CA, USA)
Toto	2017	Intermediate (±pre-atrophic changes)	15, 15	Healthy Controls	15	Superficial Retinal Plexus, Deep Retinal Plexus	Flow Density, Macular Thickness	XR Avanti AngioVue OCTA (Optovue Inc, Fremont, CA, USA)
Lee, B	2018	Early (fellow eye with Exudative AMD)	88	Healthy Fundus (fellow eye with Exudative AMD)	58	Superficial Capillary Plexus, Deep Capillary Plexus	Vessel Density, FAZ Area	DRI OCT Triton, software version 10.10 (Topcon Corp., Tokyo, Japan)
Stavrev	2018	Early, Intermediate	42, 47	Healthy Controls	66	Superficial FAZ	FAZ Area/Perimetry/Circularity	Cirrus HD-OCT, Angioplex (Carl Zeiss Meditec, Dublin, CA, USA)
Cicinelli	2018	AMD with RPD, AMD with RPD and ORA, AMD with drusen	22, 24, 22	Healthy Controls	22	Superficial Capillary Plexus, Deep Capillary Plexus	Vessel Density, FAZ Area, Retinal Layer Thicknesses	Cirrus HD-OCT 5000, Zeiss AngioPlex (Carl Zeiss Meditec, Dublin, CA, USA)
Ahn	2018	Early AMD with RPD	60	Early AMD without RPD	75	Superficial Capillary Plexus, Deep Capillary Plexus	Vessel Density, Retinal Thickness, Choroidal Thickness	Cirrus HD-OCT 5000 (Carl Zeiss Meditec, Dublin, CA, USA)
Trinh	2019	Intermediate	63	Healthy Controls	51	Superficial Capillary Plexus, Deep Capillary Plexus	Vessel Density/Length/Diameter/Complexity, FAZ Area/Perimetry/Circularity, Ganglion Cell Layer Thickness	Cirrus Angioplex OCTA (Carl Zeiss Meditec, Jena, Germany)
Reiter	2019	Intermediate	31	12 Month Follow Up	31	Superficial Capillary Plexus, Deep Capillary Plexus	Vessel Density, Flow Area, Drusen Volume	RTVue XR Avanti (Optovue, Fremont, CA, USA)
You	2020	Geographic Atrophy	10	Healthy Controls	10	Superficial Vascular complex, Intermediate Capillary Plexus, and Deep Capillary Plexus	Vessel Density, Retinal Layer Thicknesses	RTVue XR Avanti (Optovue, Inc.)
Lee, S	2020	Exudative	142	Non-Exudative AMD	168	Superficial Capillary Plexus	Vessel Density, FAZ Area/Perimetry/Circularity	Cirrus HD-OCT 5000, AngioPlex (Carl Zeiss Meditec, Dublin, CA)
Ozcaliskan	2020	Intermediate	58	Healthy Controls	62	Superficial Capillary Plexus, Deep Capillary Plexus	Vessel Density, FAZ Area, Retinal Layer Thicknesses	Topcon DRI OCT Triton (Topcon Corporation, Tokyo, Japan)
Shin	2020	Early, Intermediate and non-foveal Geographic Atrophy	83	Healthy Controls	83	Superficial Capillary Plexus	Vessel Density, Perfusion Density, FAZ Area/Perimetry/Circularity, Retinal Layer Thicknesses	Zeiss HD-OCT 5000 with AngioPlex (Carl Zieiss Meditec, Dublin, CA, USA)
Vaghefi	2020	Intermediate	34	Healthy Controls (young), Health Controls (old)	20, 21	Superficial Capillary Plexus, Deep Capillary Plexus	Vessel Density, Retinal Thickness, FAZ Diameter	Topcon DRI OCT Triton (Topcon Corporation, Japan)
Parisi	2020	Intermediate	27	Healthy Controls	20	Superficial Capillary Plexus, Deep Capillary Plexus	Vessel Density	AngioVue RTVue XR Avanti (Optovue, Fremont, CA, USA)
Qiu	2021	Exudative	30	Healthy Fellow eye, and Healthy Control	30, 30	Superficial Capillary Plexus, Deep Capillary Plexus	Vessel Density, Retinal Thickness, Retinal Nerve Fibre Layer Thickness	OCTA (Optovue, Inc., Fremont, CA, USA
Trinh	2021	Intermediate	60	Healthy Controls	60	Radial Peripapillary Capillary Plexus, Superficial Vascular Complex, Deep Vascular Complex	Vessel Perfusion	Cirrus HD-OCT v11.0.0.29946 Zeiss Cirrus Angioplex (Carl Zeiss Meditec; Jena, Germany)
Can	2022	Exudative	35	Early or Intermediate AMD (fellow eye with Exudative AMD)	35	Superficial Capillary Plexus, Deep Capillary Plexus	Vessel Density, FAZ Area/Perimetry/Circularity	RTVue XR Avanti OCT, AngioVue (Optovue, Inc., Fremont, CA, USA)

*RPD* reticular pseudodrusen, *ORA* outer retinal atrophy, *FAZ* foveal avascular zone.

### Controversies in OCTA

Vessel density measurements are not without controversy as to their use in the research setting. As shown in Table 1, a variety of different commercially available OCTA devices have been used to conduct the studies, some of which contain software that can automatically produce a vessel density reading and some that do not. Researchers whose devices lack this software must extract the images and process them using image processing algorithms, which vary in functioning and results. Furthermore, the software for each device may calculate vessel density in different ways. The Zeiss AngioPlex OCTA defines vessel density as the total length of skeletonised perfused vasculature per unit area of the measurement region whereas the Optovue AngioVue OCTA uses the total area of perfused vessels per image area [43]. Vessel density is usually the preferred OCTA metric in studies because it is comparatively simpler to calculate and less sensitive to artefacts of scan processing such as poor segmentation. More advanced metrics such as vessel diameter, length, tortuosity and fractal dimension may require a new generation of tools to accurately and reliably assess, starting with more accurate methods for segmentation mapping.

There are in fact a number of areas where lack of standardisation is an issue in OCT-A studies and this introduces problems with reproducibility and the comparison of results, particularly when comparing quantitative measurements when different settings are used. Inconsistent field of view size, methods of auto-segmentation, choice of minimum signal strength and techniques to take into account artefacts and axial length variations are all known to cause variation in results. The authors of this review strongly advise that Sampson et al.’s [43] recommendations for standardising OCTA studies are followed by future researchers in this field. Their table summarising OCTA metrics is reproduced in the supplementary material (Supplementary Table 1) for this review.

An additional area highlighted by Sampson et al. [43] that is particularly relevant for the studies included in this review is inconsistent terms used for the vascular anatomy of the retina. This can be seen in Table 1 with different studies referring to the “superficial retinal plexus”, the “superficial capillary plexus” and the “superficial vascular complex”. In part this is also due to differences in the methods that OCTA devices use for auto-segmentation of the retinal layers and the terminology that they apply to each segment. Figure 1 is reproduced from Campbell et al. [11] and neatly illustrates the commonly used terms, the retinal layers and vessel plexuses they correspond to, and an updated terminology recommended for future studies. They suggest that the four vascular plexuses can be grouped into two vascular complexes; the superficial vascular complex (encompassing the radial peripapillary capillary plexus and the superficial vascular plexus) and the deep vascular complex (encompassing the intermediate capillary plexus and the deep capillary plexus). This nomenclature is endorsed in Sampson’s recommended standards.

### Correlating vessel density with structural change

As discussed earlier (and as shown in Fig. 1), the superficial vascular plexus runs predominantly in the ganglion cell layer (GCL) of the inner retina. Accordingly, a number of studies also compared thicknesses of segmented retinal layers between patients with and without AMD, with the majority finding a significant reduction in GCL thickness in the disease group [27–30], echoing the results of previous studies [44, 45].

A number of theories have been proposed to explain this relationship and suggest a mechanism for the involvement of the inner retina in AMD. A key question is whether the changes discussed in this review are a cause, or a consequence, of the AMD disease process. Given the established record of smoking as a risk factor for AMD and to a lesser extent hypertension, obesity and diets high in saturated fat [46], it is a plausible suggestion that systemic compromise of the cardiovascular system may extend to the retinal vessels and cause reduced perfusion and hypoxia of the retinal tissues, leading to cell death in the inner retinal layers and possibly contributing to inflammation deeper into the retina. Cardiovascular disease is not however a prerequisite for AMD and as Trinh et al. point out [31], in this scenario you would expect a uniform effect on vessel density throughout the retina, whereas the results of their study and others [29] show spatial-specific reduction of vessel density.

A popular theory by Feigl et al. [47] introduced the concept of post-receptoral functional loss where neurons distal to the photoreceptors in the visual pathway are particularly susceptible to ischaemia in early AMD as they are in the “watershed” zone between the two circulations supplying retinal tissue. They suggest therefore that the bipolar cells in the inner nuclear layer are the earliest cells to be significantly affected in AMD. This has been extended by others to suggest that redundancy in other distal neurons, such as the ganglion cells, due to diminished input signal from the bipolar cells may then have a lower metabolic demand and thus require a lower blood flow rate. This process is sometimes referred to as anterograde trans-neuronal degeneration. It is not a totally satisfactory theory however as the intermediate and deep capillary plexuses are known to be situated amongst the bipolar cells whereas the majority of evidence in this review supports the superficial capillary plexus being primarily involved.

Very few studies have looked at the way in which differences in the retinal vasculature in AMD patients change over time and without this longitudinal evidence, it is difficult to know whether the observed changes contribute to, or are caused by, the pathological processes ongoing in the early stages of AMD. One approach to combining these two possible explanations is to consider a “two-hit” model whereby individuals who are at the lower end of normal variation in inner retinal blood flow (either through genetic or acquired risk factors) are at increased susceptibility of developing AMD if they also acquire an insult to their choroidal circulation, however this is conjecture and has not been investigated through research.

## Conclusion

To conclude, research into the role of the retinal vasculature has accelerated since the OCTA technology became widely available. Variability in OCTA hardware, software and imaging protocols has contributed to variability in study results however the most reliable and consistent evidence supports a reduction in the density of the vasculature in the superficial vascular complex, and possibly the deep vascular complex, in patients with early to intermediate AMD. Natural variation or the influence of systemic risk factors may predispose patients with a less dense vasculature to the development of AMD, or early pathophysiological changes may reduce the blood supply demand of the inner retinal tissues. In order to delineate this further, additional longitudinal research with a new generation of tools for analysing the vasculature is needed to monitor changes over time, ideally in prospective groups that do not have AMD at baseline. It also remains to be seen whether stratification of these vascular indices could be useful as a biomarker to help to predict progression to later stages of AMD, and therefore whether pharmacological modulation of the retinal vasculature could reduce this risk.

### Supplementary information

Supplementary Table 1: Recommended metrics for characterisation of the retinal microvascular network architecture and characterisation of foveal avascular zone based on en face OCTA images.
