# Supplementary material for: The role of the retinal vasculature in age-related macular degeneration: a spotlight on OCTA
Source: Eye (Lond). 2023 Sep 6;38(3):442–9. doi: 10.1038/s41433-023-02721-7 (PMC10858204; doi:10.1038/s41433-023-02721-7)
Supplement: Supplementary file 1 — Supplementary Table 1: Recommended metrics for characterisation of the retinal microvascular network architecture and characterisation of foveal avascular zone based on en face OCTA images. [file 41433_2023_2721_MOESM1_ESM.docx]

| **Metric (abbreviation)** | **Unit** | **Type of analyzed image** | **Definition and importance** |
| --- | --- | --- | --- |
| Vessel area density (VAD) | % | Binarized | Ratio of the area occupied by vessels (white pixels) divided by the total area converted to a percentage. The change in VAD is coupled with changes in vessel length and vessel size. Since the decrease of microvascular perfusion is sometimes accompanied by vessel dilation, VAD alone cannot provide a complete picture of changes in vessel function. Nevertheless, VAD provides the best estimate of real vessel density and can be used to demonstrate ischemia. It is recommended that VAD replaces related terms including vessel density and perfusion density. |
| Vessel length density (VLD) | % | Skeletonized | Ratio of the total length of the blood vessels to the total area. Each vessel is identified as a single pixel-width line along its centerline. VLD does not consider vessel size and compared to VAD is more sensitive to perfusion changes at the capillary level. |
| Average, median, and distribution of vessel length | mm | Skeletonized | Lengths of all identified vessel segments along the centerline of the vessel. Network interconnectivity and branching patterns can indicate oxygenation/nutrient delivery dysfunction. |
| Average, median, and distribution of vessel diameter | µm | Binarized | Diameters of all identified vessel segments. Each diameter found as the distance between edges orthogonal to centerline of blood vessels in the binarized image; each segment should be sampled at least three times. Can inform on dilation, sprouting or vessel regression. |
| Vessel diameter index (VDI) | pixels | Binarized and skeletonized | [Average vessel diameter calculated by dividing VAD by VLD; the unit is pixels and can be converted to microns by multiplying VDI by pixel size. VDI is the average vessel caliber and does not reflect the change in vessel density; therefore, VDI is sensitive to vascular dilation](https://www.nature.com/articles/s41377-022-00740-9#ref-CR154). |
| Average and distribution of vessel tortuosity (VT) | 1a | Skeletonized | [Segment length along the centerline divided by endpoint linear distance. Can inform on pathological microvascular remodeling and/or ischemia](https://www.nature.com/articles/s41377-022-00740-9#ref-CR153). |
| Branchpoint density (BD) | nodes/mm | Skeletonized | [Number of identified branchpoints divided by total vessel length. This parameter describes the level of interconnection in the microvascular network and may indicate the resilience to occlusion or blockage of blood flow](https://www.nature.com/articles/s41377-022-00740-9#ref-CR153). |
| Fractal dimension | 1 | Binarized | Calculated based on box method. Identifies the extent to which structures within the microvascular network repeat across a range of length scales. Altered spatial distribution of the capillary network is an indicator of impairment of oxygenation and nutrient delivery. |
| Non-flow area | mm2 | Binarized | [Total number of black pixels enclosed by the contour selected by the user as lacking vessels for avascular areas other than FAZ. Provides information on microvascular network defects resulting in focal decrease due to localized absence and/or collapse of retinal capillaries](https://www.nature.com/articles/s41377-022-00740-9#ref-CR150). |
| Foveal avascular zone (FAZ) area | mm2 | Binarized | Total number of black pixels enclosed by the FAZ segmentation contour. |
| FAZ perimeter length | mm | Binarized | Length of the perimeter of the FAZ. |
| FAZ axis ratio | 1 | Binarized | Ratio between the major and minor axis of the ellipse best fit to FAZ outline shape. |
| Acircularity index | % | Binarized | Ratio between the measured perimeter and the perimeter of a circular area of the same size. |
| Foveal vessel density 300 (FD-300) | % | Binarized | Percentage of the area occupied by vessels (white pixels) divided by the area in a 300-µm width rim surrounding the FAZ. The rim width value was chosen based on the relationship between FAZ and ganglion cell complex thickness in normal eyes to better distinguish between normal variations in FAZ and those due to pathology. |
| Flow area | mm2 | Binarized | Ratio of the area occupied by vessels (white pixels) divided by a region of interest chosen by the user. Used to quantify the area of flow signal within a user-predefined outer retina (vessel-free region between the outer plexiform layer and Bruch’s membrane). |

**Supplementary Table 1:** Recommended metrics for characterization of the retinal microvascular network architecture and characterization of foveal avascular zone based on en face OCTA images.

Reproduced from Sampson, D.M., Dubis, A.M., Chen, F.K. et al. Towards standardizing retinal optical coherence tomography angiography: a review. Light Sci Appl 11, 63 (2022). https://doi.org/10.1038/s41377-022-00740-9 under a Creative Commons Attribution 4.0 International License. No changes to content were made. <http://creativecommons.org/licenses/by/4.0/>
